# Prevalence of hepatitis C virus among HIV-infected patients

**Published:** 2020-04

**Authors:** Elham Zayedi, Manoochehr Makvandi, Ali Teimoori, Ali Reza Samarbaf-Zadeh, Shokouh Ghafari, Seyed Saeed Seyedian, Azarakhsh Azaran

**Affiliations:** 1 Infectious and Tropical Diseases Research Center, Health Research Institute, Ahvaz Jundishapur University of Medical Sciences, Ahvaz, Iran; 2 Department of Virology, School of Medicine, Ahvaz Jundishapur University of Medical Sciences, Ahvaz, Iran; 3 Alimentary Tract Research Center, Golestan Hospital, Ahvaz Jundishapur University of Medical Sciences, Ahvaz, Iran

**Keywords:** Hepatitis C virus, Human immunodeficiency virus, Nested polymerase chain reaction

## Abstract

**Background and Objectives:**

Hepatitis C virus and Human Immunodeficiency Virus (HIV) share the same rate of transmission. HIV/HCV co-infected individuals may result in faster progression of liver fibrosis and highly increase the risk of cirrhosis, hepatocellular carcinoma development. Thus this study was conducted to determine co-infection of HCV genotypes in positive HIV patients in Ahvaz city, Iran.

**Materials and Methods:**

The sera samples were collected from confirmed 78 infected HIV, 67 (85.89%) males and 11 (14.1%) females. All sera samples were tested for HCV Ab using ELISA test. The HCV Ab positive samples were tested for detection of 5′ untranslated (UTR) and core regions of HCV genome using nested RT-PCR. The PCR products of 5UTR and core regions were sequenced to determine HCV genotypes.

**Results:**

Among the 78 infected HIV, 25 (32.05%) cases including 20 (25.64%) males and 5 (6.41%) females were positive for HCV Ab (*p*=0.316). 53 (67.94%) of HIV patients were negative for HCV Ab. Among 25 positive HCV Ab, 19 (24.35%) cases including 15 (19.23%) males and 4 (5.12%) females were positive for HCV RNA (*p*=0.447). The PCR products of 5 positive samples were randomly sequenced. The results of sequences and alignments showed that the detected HCV genotypes were three 3a and two 1a. The occurrence of genotype HCV 1a was found in one male injecting drug user Injecting Drug User (IDU) and one female. The HCV 3a genotype was detected in the three males IDU.

**Conclusion:**

The results of this survey indicated that 32.05% of HIV patients were positive for HCV Ab, among them 24.35% were positive HCV RNA. HCV genotype 3a was dominant and detected in the three males IDU. Regarding the consequences of HIV/HCV co-infection, it is suggested that HCV RNA detection should be regularly checked in individuals infected with HIV.

## INTRODUCTION

Hepatitis C Virus (HCV) is a single-stranded, positive-sense RNA virus belonging to the genus hepacivirus and family Flaviviridae (1). Approximately, 177.5 million of the world population suffer from chronic HCV infection (2). Long term persistence chronic HCV infection may result in cirrhosis, hepatic failure, or hepatocellular carcinoma (HCC), which are responsible for approximately 350000 to 500000 deaths per year worldwide (3). Moreover, long-term HCV chronic infection result in some extrahepatic manifestations with serious consequences, such as cryoglobulinemia, porphyria cutaneous tarda (PCT) disease, type 2 DM, thyroid disorders, glomerulonephritis, and lichen planus (4). HCV is transmitted through blood transfusion, injecting drug use (IDU), sexual intercourse, surgery, and tattooing (5, 6).

HCV has been classified into 7 genotypes (1–7) and more than 100 subtypes (7). The distribution of HCV genotypes varies in different regions of the world. HCV genotypes 1 and 2 are most common in North America, Japan and Europe whereas HCV genotype 3 is predominant in South-east Asia and India. HCV genotype 4 is the most common genotype in Middle Eastern countries such as Egypt, Syria and Saudi Arabia. HCV genotype 5 is common in South Africa (8). The high genetic diversity of HCV poses an obstacle for vaccine development and impedes effective antiviral therapy (9). In Iran, the prevalence of HCV in the general population and high-risk populations is 0.3% and 32.1% respectively (10). The HCV genotypes 1a, 3a, 1b are the most dominant in different regions of Iran (7, 11). Due to sequence diversity and variation of hepatitis C genome, no vaccine is available against HCV infection. No decisive antiviral therapy is available against HCV infection even through recent direct acting antiviral (DAA) therapy (12).

Human immunodeficiency virus (HIV) is the Lentivirus genus and belongs to the Retroviridae family. About 37.9 million [32.7 million–44.0 million] people globally were living with HIV by the end of 2018 (13). HIV-1 is circulating in Iran (14). HIV individuals are at risk of acquiring HCV infection because both viruses infect people via sexual intercourse, intravenous drug use, blood transfusions and from mother to her baby (15). Co-infection with HIV/HCV develop a higher rate of liver fibrosis, cirrhosis, hepatocellular carcinoma and death (16–18). Due to, high genetic diversity of HIV, no vaccine and decisive treatment are available against HIV infection (19, 20). Since HIV individuals are at high risk of HCV infection, therefore this study was conducted to evaluate the prevalence of HCV among the infected individuals with HIV.

## MATERIALS AND METHODS

### Ethic consent.

This study was approved with registration number IRAJUMS.REC 1394513 by the ethics committee of Ahvaz Jundishapur University of Medical Sciences, Ahvaz, Iran. All experiments were performed in compliance with relevant laws and institutional guidelines and in accordance with the ethical standards of the Declaration of Helsinki. The ethic consent was obtained from each participant registered in this study.

### Study design and population.

The sera were collected from 78 new confirmed positive HIV patients including 67 (85.89%) males and 11 (14.1%) females who have attended for HIV treatment in Razi Hospital, Ahvaz city, during February to November, 2017. The inclusion criteria was patient’s age ≥ 20 years, patients who was willing to take part in the study. Patients who were not willing to take part in the study were excluded. CD4 cell counts for HIV patients were analyzed using the Becton Dickinson (BD) FAS Count system (Becton, Dickinson, USA).

The sera of all participants were tested for, alanine transaminase (ALT), and aspartate transaminase (AST) using commercial kits (Bionic, Iran). The sera were also tested for HCV antibodies using ELISA according to manufacturer instructions (Diapro, Italy). The seropositive HCV samples were further tested for amplification of the 5′-untranslated (5′UTR) and core regions of HCV genome by nested reverse transcriptase-polymerase chain reaction (RT-PCR).

### RNA extraction and cDNA synthesis.

Total RNA was extracted from all precipitants sera using high pure viral nucleic acid kit (Roche Diagnostics GmbH, Mannheim, Germany) according to the manufacturer’s instruction. cDNA was synthesized from the extracted RNA using Thermo scientific (Latvia) according to manufacturer instruction. The prepared cDNA was used for amplification of 5′UTR and core regions of HCV genome using nested RT-PCR.

### Nested RT-PCR for 5′ untranslated region (UTR).

The following primers, BKP-7,-CACTCCCCTGTGAGGAACTACTGTC (nucleotides 38 to 62) as the outer sense, BKP-8,ATGGTGCACGGTCTACGAGACCTCC (nucleotides 319 to 343) as the outer anti-sense; BKP-9,TTCACGCAGAAAGCGTCTAGCCATG (nucleotides 63 to 87) as the inner sense; BKP-10,GCGCACTCGCAAGCAC-CCTATCAGG (nucleotides 292 to 314) as the inner anti-sense primer were used for detection of 5′UTR region of Hepatitis C virus genome using nested RT-PCR: (21). For the first round, the reaction mixture containing 2.5 μl 10× PCR buffer with MgCl _2_ (Roche), 0.5 μl dNTP mix (0.2 mM), 1 μl of BKP-7 and BKP-8 primers (10 pmol), 0.2 μl (1 unit) Taq polymerase, 5 μl of template and water up to 25 μl. The PCR was performed on Techne thermocycler (Techne, UK). The cycling conditions were as follows: 94 °C for 4 min; 35 cycles at 94 °C for 30 sec, 60 °C for 30 sec and 72 °C for 30 sec with a final extension at 72 °C for 10 minutes. The second round was carried out like the first round with the inner set of primers (BKP-9 and BKP-10) with the same PCR mixture and program. 3 μl of first round product was used as template for the second round PCR. PCR product was subjected to electrophoresis on a 2% agarose gel, stained with DNA safe stain, and observed under ultraviolet light. The first round PCR product was 306 bp for the outer set and the second product was 254 bp for the inner set.

### Nested RT-PCR for core region.

The samples positive for 5’UTR region were tested again for HCV core region of the HCV genome by nested RT-PCR. The following primers, including outer Forward, SC2: GGGAGGTCTCGTAGACCGTGCACCATG, outer Reverse, AC2: GAGMGGKATRTACCCATGAGRTCGGC, Inner Forward S7:AGACCGTGCACCATGAGCAC and 584: CCCATGAGGTCGGCRAARC were used for amplification of HCV core region of HCV genome (22). Briefly, 3 μl of the template with the same amount of PCR reaction mixture as described previously, was subjected to thermal cycler for 30 cycles which was used for the first and second runs. The cycling conditions were as follows: 94 °C for 4 min; 30 cycles at 94 °C for 1min, 45 °C for 1 min, 72 °C for 2 min, and final elongation at 72 °C for 7 min. PCR product was subjected to electrophoresis on a 2% agarose gel, stained with DNA safe stain, and observed under ultraviolet light. The expected PCR products for the outer set and the inner set were 500 bp and 420 bp, respectively.

### HCV genotyping/subtyping.

The PCR products of 5 samples positive for 5’UTR and core regions were sequenced (Bioneer Company, South Korea). The sequences 5’UTR and core regions were sent to GenBank to obtain accession number.

### Phylogenetic analysis.

The phylogenetic tree for 5UTR and core regions of the HCV genome was constructed using Maximum likelihood method. The results of sequences of HCV 5UTR and core regions of HCV genome isolated from Ahvaz city were compared with other different HCV genotypes and subtypes isolated from different regions of the world. The Maximum likelihood method was done under parameters including the site heterogeneity gamma and invariant sites, phylogenetic distances by Kimura two-parameter model using MEGA 6 software (https://www.megasoftware.net/). The scale bars represented the frequency of nucleotide substitutions. The accuracy of the tree was assessed by 1000 bootstrap replicates.

### Statistical analysis.

The statistical analysis was performed using SPSS (Version 19) software to compare variables such as gender, age, liver function test, CD4 count, and distribution of HCV Ab and HCV RNA detection among the gender using qua-square test, and t-student test. P value >0.05 was considered as the level of significance.

## RESULTS

A total number of 78 HIV patients, 67 (85.89%) males and 11 (14.1%) females, were participated in this study. The patients’ age ranged from 20 to 51 with the mean age of 33.04 ± 6.207 years. CD4 count was 261–648/μl. The level of ALT (50–175 IU/L) and AST (45–112 IU/L) were raised among all the patients with HIV/HCV co-infection. Overall, 25 (32.05%) patients including 20 (25.64%) males and 5 (6.41%) females were positive for anti-HCV antibody (*p*=0.316). Among 25 positive HCV Ab, 19 (24.35%) cases including 15/67 (22.38%) males and 4/11 (36.36%) females were positive for HCV RNA (*p*=0.447). 53 (67.94%) of HIV patients were negative for HCV Ab. Table 1 shows the profile of patients.

**Table 1.** Summary of patients’ demographic information.

**Characteristics**	**(n=78)**
Median age, years	29 ± 3
Gender	
Male	67 (85.9%)
Female	11 (14.1%)
Marital status	
Single	32 (41%)
Married	41 (52.6%)
Divorced/separated	3 (3.8%)
Widow	2 (2.6%)
Occupation	
Unemployed	37 (47.4%)
Self-employed	30 (38.5%)
Employed	1 (1.3%)
Housewife	10 (12.8%)
Literacy	
Primary school	53 (68%)
Secondary school	24 (30.7%)
Diploma	1 (1.3%)
University degree	--
Risk factors	
IDU	65 (83.3%)
High risk sexual behavior	63 (80.7%)
Heterosexual	59 (75.6%)
Male-to-male sexual contact	11 (14%)
Tattoo	48 (61.5%)
Prison record	62 (79.5%)
CD4 cell count	261–648/μl
HAART	17 (21%)
ALT	50–175 IU/L
AST	45–112 IU/L
History of blood transfusion	0 (0)

Six HIV patients including 5 (6.41%) males and 1 (1.28%) female were positive for HCV Ab but negative for HCV RNA. High prevalence of 16/65 (21.53%) HCV RNA was observed among the IDU followed by prison record 8/62 (12.9%), high risk sexual behavior 5/63 (7.93%), heterosexual 4/59 (6.34%), male-to-male sexual contact 1/11 (9.09%) and tattoo 3/48 (6.25%). The distribution of HCV-RNA among different age-group of patients with HIV exhibited that the higher frequency of 12.64% HCV-RNA were observed in the age-group 30-39 years and the lower frequency of 1.14% of HCV-RNA were found in the age-group >50 (*p*=0.810). Table 2 shows the results of frequency of HCV-RNA detection among the different age-group of HIV patients.

**Table 2.** Distribution of HCV infection among different age-groups.

**Age-group**	**HCV-RNA** **Negative No: 59**	**HCV-RNA** **Positive No: 19**	**P value**
< 20	---	---	
20–29	5 (5.74%)	3 (3.44%)	
30–39	35 (18.39%)	11 (12.64%)	0.810
40–49	16 (18.39%)	4 (4.59%)	
> 50	3 (3.44%)	1 (1.14%)	

### Sequencing.

**Fig. 1.**
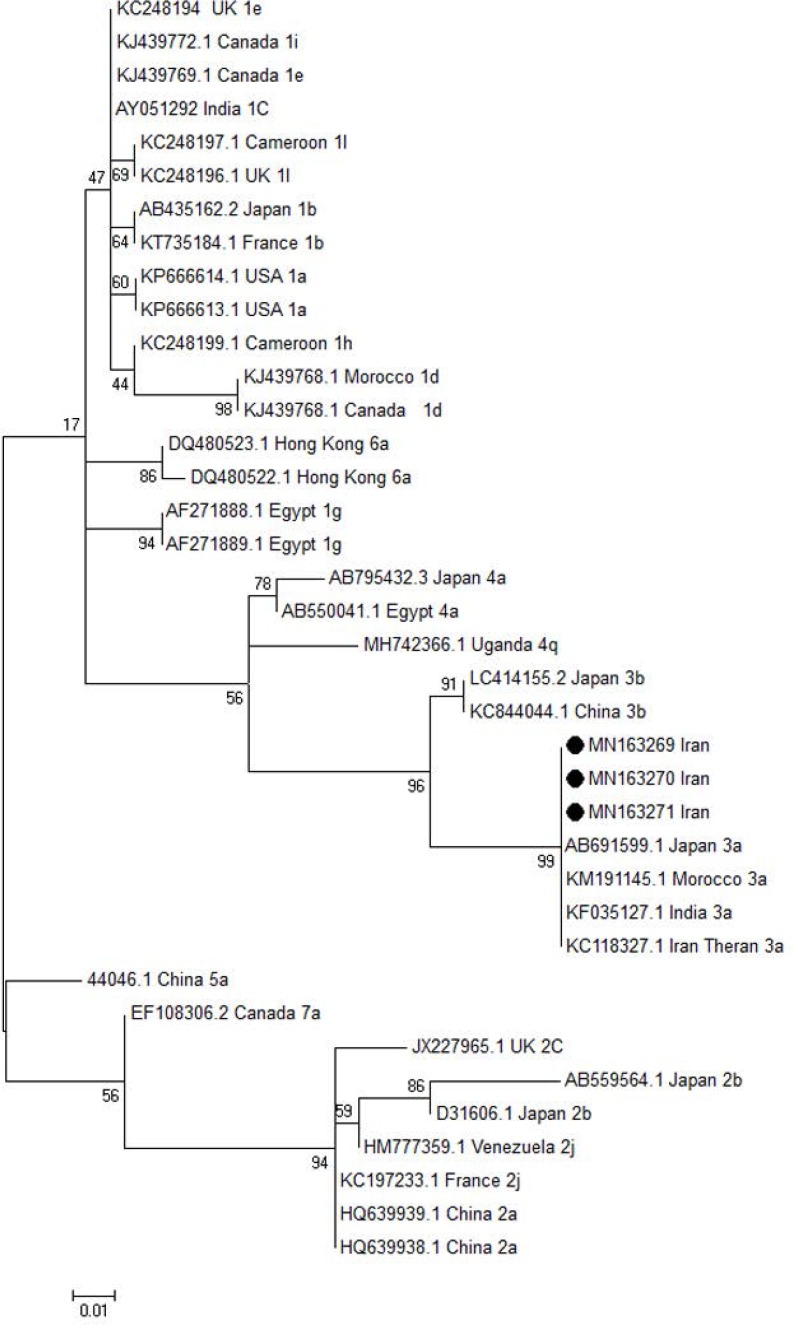
The phylogenic tree was constructed for 5UTR region of HCV genome isolated from Ahvaz, Iran. The detected HCV 5UTR region with accession numbers MN163269-71 was compared with different HCV genotypes and subtypes retrieved from GenBank. The maximum likelihood tree method was done under parameters including with the site heterogeneity gamma and invariant sites, phylogenetic distances by Kimura two-parameter model using MEGA 6 software. The Iranian isolated HCV 5UTR region with black circle were clustered with HCV genotype 3a isolated from different regions of the world. The accuracy of the tree was assessed by 1000 bootstrap replicates. The scale bars is equal to 0.01.

**Fig. 2.**
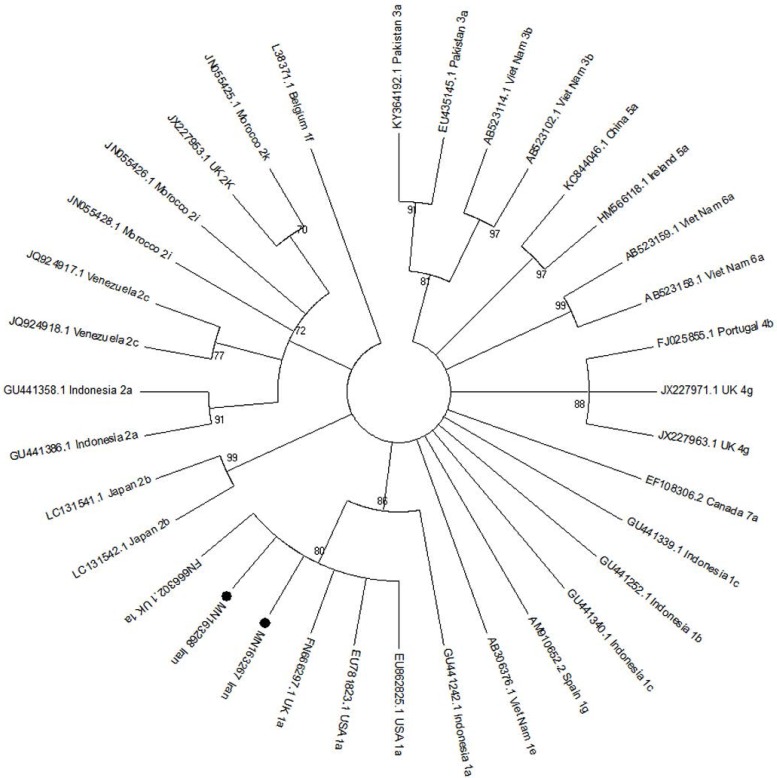
The phylogenic tree was constructed for core region of HCV genome isolated from Ahvaz, Iran. The detected HCV core region with accession numbers MN163267 and MN163268 were compared with different HCV genotypes and subtypes retrieved from GenBank. The maximum likelihood method was done under parameters including with the site heterogeneity gamma and invariant sites, phylogenetic distances by Kimura two-parameter modelusing MEGA 6 software. The Iranian isolated HCV core region with black circle were clustered with HCV genotype 1a isolated from different regions of the world. The accuracy of the tree was assessed by 1000 bootstrap replicates. The scale barsis equal to 0.01.

The results of sequencing and alignment of the two detected core region showed HCV genotype 1a. The results of sequencing and alignment of the three isolated 5UTR region showed HCV genotype 3a. The sequences were recorded in the GenBank accession numbers MN163267, MN163268 for detected HCV core and three accession numbers MN163269-71 were recorded for HCV 5UTR samples. The occurrence of HCV genotype 1a was found in one male injection drug use (IDU) and one female. The HCV 3a genotype was detected in the three male IDU. The results of phylogenic tree for 5 UTR region of HCV genome revealed that the three isolated HCV (MN163269-71) are cluster with HCV genotype 3a (Fig. 1). The results of phylogenic tree for core region of HCV genome showed that the two isolated HCV (MN163267,MN163268) are cluster with HCV genotype 1a (Fig. 2).

## DISCUSSION

Evaluation of the HCV and HIV co-infection is crucial for prognosis and treatment of both viruses. Precise observation can expedite the treatment of chronic HCV infection and will help to prevent cirrhosis, end-stage liver disease, and liver carcinoma development.

In the present survey, 19 cases (24.35%) of the HIV patients including 15 (19.23%) males and 4 (5.12%) females were positive for HCV RNA (*p*=0.447). Kinkel et al. studied 397 participants with known HIV-status and detected 72 (18.1%) cases of HIV/HCV co-infection (HCV-RNA and anti-HIV-antibodies) which was congruent with our results (23).

The intravenous drug user are at risk of various factors such as narcotics, tattooing, physical abuse, lack of proper health facilities, and lack of social and family support; hence, they can be easily exposed to HCV (24).

In our survey, high prevalence of HCV RNA/HIV co-infection were found among the IDU 16/65 (21.53%), followed by prison record 8/62 (12.9%), high risk sexual behavior 5/63 (7.93%), heterosexual 4/59 (6.34%), male-to-male sexual contact 1/11 (9.09%) and tattoo 3/48 (6.25%). Malekinejad et al. reported the prevalence of HCV/HIV co-infection among the injecting drug user (IDU) was 11.3% in Tehran, Iran (25).

Frequency of HCVgenotypes have been reported among the HIV patients in the different regions of the world. In our present survey, HCV genotype 3a and genotype 1a were dominant among the HIV patients. Rossetti et al. have detected a high prevalence of HCV genotypes 1a and 3 among the positive HIV patients in Italy (26). The occurrence of genotype HCV 1a was found in one male IDU and one female. All three HCV 3a genotype were found in the three males IDU. Robaeys et al. in Belgium have reported the HCV genotype 3a and 1a were dominant in people who inject drugs (PWID) (27).

Occult hepatitis C virus (HCV) infection (OCI) is defined as the presence of HCV RNA in hepatocytes or peripheral blood mononuclear cells (PBMCs) with no detectable HCV RNA in the serum. Two types of OCI have been classified: seronegative OCI (anti HCV antibody-negative and serum HCV RNA-negative); and, seropositive OCI (anti-HCV antibody-positive and serum HCV RNA-negative) (28).

In this study 6 HIV patients including 5 (6.41%) males and 1 (1.28%) female were positive HCV Ab but negative for HCV RNA. The occurrence of occult hepatitis C virus (OCI) have been reported among the individuals infected with HIV in Tehran, Iran (29). In the present study the occurrence of OCI have not been investigated among the HIV patients.

The arrival of a notable direct-acting antivirals (DAAs), the treatment of HCV infection has been greatly revolutionized. High efficacy HCV regimens such as sofosbuvir (SOF) plus ribavirin (RBV), SOF plus daclatasvir (DCV), SOF plus ledipasvir (LDV), ritonavir-boosted ombitasvir plus paritaprevir (2D) ± dasabuvir (3D), grazoprevir (GZV) plus elbasvir (EBV), SOF plus velpatasvir (VEL) or glecaprevir (G) plus pibrentasvir (P) have been reported in HCV/HIV co-infected patients (30, 31).

Early diagnosis and treatment are crucial for curbing hepatitis C virus infection in different high risk group. On the other hand, patients co-infected with HIV/HCV develop a higher rate of liver fibrosis, cirrhosis, hepatocellular carcinoma and death (16–18). Thus, to manage the better treatment outcomes, screening of the HCV RNA by molecular means should be implemented for HIV patients periodically.

In conclusion, the results of this survey indicated that 32.05% of HIV patients were positive for HCV Ab, among them 24.35% were positive for HCV RNA. HCV genotype 3a was dominant in IDU. All the co-infected HIV/HCV have raised abnormal ALT. Six HIV patients including 5 (6.41%) males and 1 (1.28%) female were positive for HCV Ab but negative for HCV RNA. The determination of OCI was not investigated in HIV patients. Concerning the consequences of HCV/HIV co-infection, to prevent HCV infection, to manage and improve treatment outcomes, screening of HCV RNA should be regularly checked in individuals infected with HIV.
